# Can work climate foster pro-environmental behavior inside and outside of the workplace?

**DOI:** 10.1371/journal.pone.0223774

**Published:** 2019-10-10

**Authors:** Carol Hicklenton, Donald W. Hine, Natasha M. Loi

**Affiliations:** School of Psychology, University of New England, Armidale, New South Wales, Australia; Middlesex University, UNITED KINGDOM

## Abstract

Guided by self-determination theory, we investigated the potential impact of work climate on employee motivation, and pro-environmental behavior (PEB) inside and outside of the workplace. We found that in workplaces with stronger pro-environmental climates and at least moderate levels of autonomy support, employees reported higher levels of autonomous motivation to engage in PEB. In turn, autonomously motivated employees engaged in more PEBs, both inside and outside the workplace. Controlled motivation played a more limited role in predicting employee PEBs. Overall, our findings suggest work climates that support pro-environmental actions and employee autonomy may not only foster PEBs within the workplace but also lay the foundation for PEBs in other non-workplace settings.

## Introduction

Organizations are increasingly adopting pro-environmental policies and procedures. Annual reporting on environmental management and carbon emission reduction, by the largest companies based on revenue, increased from 44% in 2011 to 78% in 2017, and from 58% in 2015 to 67% in 2017, respectively [1]. Regardless of whether these shifts were driven primarily by regulatory pressure, a desire for cost savings, or reputation concerns, it is important to recognize that the successful implementation of “green” policies and procedures in corporate environments requires not only pronounced pro-environmental work climates but also the cooperation of individual employees.

Work climate involves employees’ perceptions of the organization’s environment and its priorities. Although there is growing evidence that pro-environmental work climate may be an important driver of employee pro-environmental behavior (PEB), the precise mechanisms by which climate exerts its effects remain unclear. One promising avenue of research involves investigating how work climate influences employee motivation for engaging in PEB [2]. In this study, we used self-determination theory (SDT) to investigate the potential effects of employees’ perceptions of two types of work climate on employees’ motivation to engage in PEB: (1) the extent to which organizations actively encourage pro-environmental outcomes through their policies, processes, and practices (pro-environmental climate), and (2) the extent to which they support employees’ autonomy for work tasks (autonomy support). We also explored the role of employees’ autonomous and controlled motivation for PEB as mediators of relationships between work climate and PEB. Finally, we explored whether certain types of work climate can elicit employee motivational patterns that are conducive to positive spillover effects, in which employees also engage in PEBs outside the workplace.

### 1.1 Self-determination theory

According to SDT, humans are “naturally inclined to act on their inner and outer environments, engage in activities that interest them, and move toward personal and interpersonal coherence” [3]. SDT distinguishes between two types of motivation: *intrinsic* and *extrinsic*. Intrinsic motivation is fully autonomous. Intrinsically motivated activities are done because they are interesting or enjoyable in themselves. In contrast, extrinsic motivation regulates activities done as a means to an end. SDT specifies four main types of extrinsic motivation that vary based on whether the source of the regulation of a behavior is more or less internalized, that is self-regulated [3].

1. *External regulation* is the most controlled form of extrinsic motivation. It occurs when behavior is “regulated by others’ administration of contingencies” [3]. For example, in the context of a proactive pro-environmental organization, external regulation could arise if employees feel that bonuses or promotion depend on complying with the company’s waste reduction policies and procedures.
2. *Introjected regulation* is a controlled form of internal regulation. This form of control is manifested via individuals being motivated to act out of feelings of pressure from the self which then results in a sense of obligation to do a given behavior [3]. For example, some employees may comply with waste reduction policies to avoid feeling guilty rather than being guided by any expectation that they would be rewarded for complying or punished for not complying.
3. *Identified regulation* occurs when people recognize and accept the underlying value of a behavior, but have not yet fully integrated the behavior with other aspects of their identity [3]. This might occur when employees believe, in general, that it is important to minimize waste, and, as a consequence, use the recycling bins at work most of the time. But there are still instances, such as when recycling bins are not easily accessible, that they will not engage in the behavior.
4. *Integrated regulation* is considered the most complete expression of internalized extrinsic motivation given that it involves not only identifying with the importance of behaviors, but also ensuring those identifications become integrated with other aspects of the self [3]. Employees guided by integrated regulation view their own environmentally friendly actions as a core part of their identity.

SDT scholars often group intrinsic motivation with integrated regulation and identified regulation into a general category labelled *autonomous motivation*, given that all three types of motivation involve a high degree of internalization and volition. Similarly, introjected regulation and external regulation are often combined into a general category called *controlled motivation*, given that behavior is regulated by consequences administered by others (e.g., external rewards such as bonuses) or by individuals to themselves (e.g., feelings of guilt).

### 1.2 Pro-environmental climate and employee PEB motivation

Work climate refers to policies, practices, and procedures that guide employee behavior by indicating an organization’s priorities. Whereas, work *culture* reflects the broad assumptions and values of an organization, work *climate* reflects more tangible aspects of working environments such as specific policies, processes, and practices [4]. In the current study, pro-environmental climate is assessed via individual employees’ perceptions of the extent to which their organization acts to protect the environment. This is a form of psychological climate [5].

Most organizations have multiple work climates that operate simultaneously and help employees, motivated to succeed, to understand which behaviors they are expected to perform and why [6]. For example, a strong safety climate has been associated with fewer work accidents and a strong service climate with higher customer satisfaction [7]. Similarly, research suggests that organizations with strong pro-environmental work climates report higher rates of employee PEB [8]. For example, Kim et al. found that employees engaged in more PEB when they perceived that environmental management was integrated with HR processes such as training and performance appraisals [9]. Ruepert et al. demonsrated how workplaces with pro-environmental climates can activate social norms that encourage workers to engage in PEB [10]. In addition, Norton et al. reported that employees engaged in more PEB when they considered their organization to be committed to pro-environmental practices and also when they saw their colleagues engaging in PEB [2].

### 1.3 Autonomy support and employee motivation

Research indicates that social contexts can function to either support autonomy or control behavior [11]. Higher levels of autonomy support, relative to more controlling work environments, have been linked to a range of positive outcomes including task engagement, creativity, and behavior change [12]. Pelletier et al. noted that most policies—whether originating from government or from companies themselves—represent explicit attempts to control behavior and, as such, may sometimes be counterproductive in the long run if they too strongly reinforce controlled, as opposed to autonomous, motivation [13]. Generally, controlled motivation is associated with unstable behavior change because individuals have not internalized the regulations controlling the behavior. For example, when workplace PEB is reinforced by an organization’s employee recognition program, the target behaviors typically last only as long as the intervention. When the contingencies supporting policy adherence are removed, behavior reverts back to a more heterogeneous non-constrained state. In other words, when policymakers adopt policies that rely on reward and punishment (producing controlled motivation), they are also undertaking a long-term commitment of policing behavior which can be both inefficient and time-consuming.

It is worth noting that workplace rewards, deadlines, or positive feedback can be experienced as either autonomy supportive or controlling depending on the interpersonal context [11]. Interpersonal conditions that offer choice, explain reasons behind demands and rules, are aware of people’s feelings, and accept other points of view are more likely to generate autonomous motivation for a task by supporting individuals to explore issues and options for themselves and to choose to act in ways that are personally meaningful. SDT studies found employees in work environments with high levels of autonomy support report higher levels of autonomous motivation for work and higher levels of work satisfaction [14]. Furthermore, training managers to be more autonomy supportive produces a range of positive work-related employee attitudes such as trust in the corporation and management [11].

### 1.4 Autonomous and controlled motivation and PEB

SDT workplace studies have shown that employees’ reasons for putting effort into their jobs predicts workplace performance [12]. Specifically, employees who report doing their job because it is enjoyable or because it aligns with their interests and values (i.e., autonomous motivation) tend to be more proficient and adaptive in their jobs, often expending extra time and effort at work. On the other hand, employees who report doing their job to make a lot of money, or because their reputation depends on it (i.e., controlled motivation) are more likely to exhibit diminished vitality at work, feel less able to cope with workplace change, and put less effort into their jobs [12].

SDT studies outside the workplace have also shown that autonomous motivation is a positive predictor of PEB. Pelletier et al. found that auonomously motivated people reported engaging in a broader range of PEBs, more difficult PEBs, and sustained engagement in PEBs [15]. Pelletier et al. found respondents engaged more frequently in a broad range of PEBs if they found the PEB pleasurable (intrinsic motivation), believed the PEB was a fundamental part of who they are (integrated regulation), or they believed the PEB was an important or sensible thing to do (identified regulation). In contrast, high levels of controlled motivation only weakly predicted PEB frequency; in some instances, opposite to the expected direction [16].

In a study investigating intended effort and attainment of personal goals, Sheldon et al. found that participants who pursued their personal goals for autonomous reasons were more likely to report that they were still investing effort in their goals 8 and 15 weeks later. They also reported higher levels of goal attainment [17]. In contrast, participants who described their goals as being controlled by internal pressure (e.g., guilt) or external pressure (e.g., desire for recognition) reported diminishing motivation over time and less goal attainment [17]. According to Koestner et al. autonomous goals are “protected and maintained in the face of task-irrelevant temptations because they are continually energized” by the self [18]. Overall, these studies support the conclusion that individuals with increased autonomous PEB motivation engage in more PEB.

### 1.5 Spillover to non-workplace PEB

In a recent review of the literature, Truelove et al. proposed a framework for when PEB spillover effects are most likely to occur and not occur [19]. The framework suggests that spillover from a role-related behavior is most likely to occur when: (1) PEB is internally (as opposed to externally) motivated, (2) the behaviors in the primary and spillover domains are similar to each other, and (3) the spillover behaviors are relatively easy to perform. Of particular relevance to the present study is the internalization of motivation for workplace behaviors. According to SDT, internalization is an active and natural process of socialization in which individuals attempt to transform external regulations into personally endorsed values and self-regulations [3]. As such, changes in the quality of a person’s motivation may explain positive spillover effects to other contexts.

### 1.6 Current study

This study investigated the potential impact of two aspects of work climate (i.e., pro-environmental climate and employee autonomy support) on employees’ autonomous and controlled motivation for engaging in PEB. The study also explored the extent to which these two types of motivation predicted employee PEB both inside and outside of the workplace. Based on our review of the literature, we predicted that in organizations with strong pro-environmental climates and strong support for worker autonomy, employees would report higher levels of autonomous motivation to engage in PEB. In turn, we expected employees with higher levels of autonomous PEB motivation would be more likely to engage in more PEB both inside and outside the workplace. We also predicted that in organizations with strong pro-environmental climates but low autonomy support, employees would report higher levels of controlled PEB motivation. In turn, employees with higher controlled motivation for PEB were expected to engage in more workplace, but not non-workplace, PEB. That is, we expected autonomous motivation for PEB to produce spillover effects outside of the workplace, whereas we expected controlled motivation for PEB to guide workplace behaviour only, and not spillover to other contexts.

## Methods

### Participants

A sample of 818 Australian adults participated in this study. All were employed full time when they completed the survey. Participants were recruited from a Qualtrics^™^ research panel, and received a small monetary payment for completing the survey. Women accounted for just over half the sample (52%). Ages ranged from 18 to 69 years: 18–24 (8%), 25–34 (35%), 35–44 (29%), 45–54 (16%), 55–64 (11%), and 65+ years (<1%). The sample included a range of education levels: less than year 10 (<1%), year 10 high school (5%), year 12 high school (15%), vocational education training certificate (17%), diploma or advanced diploma (14%), graduate diploma or bachelor degree (34%), postgraduate university degree (15%). The survey was developed using the Qualtrics^™^ online survey platform (Provo, UT). The project was reviewed and approved by the University of New England (UNE) Human Research Ethics Committee.

### Measures

The survey consisted of measures assessing employee perceptions of workplace pro-environmental climate and autonomy support, motivations to engage in PEB, and frequency of workplace and non-workplace PEB. The survey also included measures of employee pro-environmental attitude, needs satisfaction, work withdrawal, and job satisfaction, which were used for a separate study.

#### Work climate

Two aspects of work climate were assessed: pro-environmental climate and employee autonomy support. Employees’ perceptions of their organizations’ commitment to positive environmental outcomes (i.e., pro-environmental climate) were assessed using the Green Work Climate Perceptions Scale [2]. The scale comprised four items, including: “Our company is worried about its environmental impact” and “Our company believes it is important to protect the environment”. Participants indicated their agreement with each statement on a scale from 1 (*strongly disagree*) to 5 (*strongly agree*). Cronbach’s alpha for the scale was .92, indicating high internal consistency.

Employee autonomy support was assessed using the Perceived Autonomy Support Scale [12, 14, 20]. This 9-item scale measures employees’ perceptions that their supervisors (i.e., immediate line managers, superiors, or more experienced professionals in a supervisory role) encourage their self-determination and autonomy within the workplace by offering choice (e.g., “My supervisors give me many opportunities to make decisions in my work”), explaining reasons behind demands and rules (e.g., “When my supervisors ask me to do something, they explain why they want me to do it”), and acknowledging their feelings (e.g., “My supervisors are open to my opinions and my point of view regarding work even when they are different from theirs”). All responses were measured using a 7-point scale (1 = *do not agree at all*, 7 = *very strongly agree*). Cronbach’s alpha for the scale was .94.

#### Motivations for pro-environmental behaviour

Employees’ motivations for engaging in PEB were assessed by two subscales of the Motivation Toward the Environment Scale [16]. Autonomous motivation was assessed by 12 items addressing intrinsic or more internalized reasons individuals may have for engaging in PEB. Four items each that reflect intrinsic motivation (e.g., “For the pleasure I experience while I am mastering new ways of helping the environment”), integrated regulation (e.g., “Because being environmentally-conscious has become a fundamental part of who I am”), and identified regulation (e.g., “Because it is a sensible thing to do in order to improve the environment”). Controlled motivation was assessed by eight items addressing extrinsic reasons for engaging in PEB. Four items each that reflect introjected regulation (e.g., “Because I would feel guilty if I didn’t”) and external regulation (e.g., “To avoid being criticized”). Both subscales were measured on a 7-point scale (1 = *does not correspond at all*, 7 = *corresponds exactly*). Cronbach’s alpha was .94 for autonomous motivation and .79 for controlled motivation.

#### Workplace pro-environmental behaviour

Workplace PEB was assessed using a 20-item frequency measure [21] describing behaviors that can be performed by participants in relation to their work (e.g., “I wear more clothes instead of putting the heating on”, “I print double-sided”, and “When I purchase goods or services, I pay attention to sustainability”). Minor changes were made to three items to better fit the Australian context. The original 6-point frequency scale ranged from 1 (*N/A*; not applicable) to 2 (*never*) through to 6 (*always*). In the current study, the scale was recoded as follows: N/A = (missing data), 1 = (*never*) to 5 = (*always*) to reflect that some of the activities, such as adjusting the office heating, were likely beyond the control of the participants. In the analysis, the mean was computed for each participant based only on the items within their control. Cronbach’s alpha for the scale was .86.

#### Non-workplace pro-environmental behaviour

Non-workplace PEB was assessed with the 31-item Frequency of Conscious Environmental Behavior Scale [22]. Participants indicated how often they engaged in activities (e.g., “Buy products that do not damage the environment” and “Recycle glass jars/bottles”) using a 7-point scale ranging from 1 (*never*), 4 (*about half the time*) to 7 (*always*). In addition, seven of the original items were modified to better reflect Australian terminology and practices (e.g., “gasoline consumption” was changed to “petrol consumption”; “Reuse paper lunch or grocery bags” was changed to “Reuse shopping bags”). Contact the lead author for details. Cronbach’s alpha for the scale was .95.

#### Ecological worldview

Ecological worldview was included as a covariate in all analyses to control for common method bias [23] and also the possibility that pre-existing differences in environmental orientation might confound the effects of pro-environmental work climate on motivation and PEB. Worldview was assessed using the revised New Ecological Paradigm (NEP) scale [24]. The scale consists of 15 items reflecting both environmental concern and beliefs that humans can dominate nature (e.g., “When humans interfere with nature it often produces disastrous consequences” and “Humans will eventually learn enough about how nature works to be able to control it”). Participants indicated the extent they agreed with each statement using a 7-point scale ranging from 1 (*strongly disagree*) to 7 (*strongly agree*). High NEP scores reflect environmental concern, defined as recognition that the earth’s carrying capacity is limited and that we are rapidly approaching these limits. Low NEP scores reflect an anthropocentric worldview, defined as believing that the earth’s resources should be exploited for human benefit and that our ingenuity as a species will enable us to overcome environmental problems as they arise. Cronbach’s alpha was .82.

### Statistical analyses

All statistical analyses were conducted using SPSS (Version 25). Mediation and moderation tests were conducted using the PROCESS macro [25]. To control for the large number of statistical tests computed, we adopted a conservative critical *p*-value of .01 and 99% confidence intervals. The survey used a forced response format, so there were no missing data. Examination of boxplots revealed a small number of univariate outliers on most of the variables included in the model, but no extreme scores. Eight multivariate outliers were identified, and the analyses were re-run with the outliers removed. Our analyses generated the same substantive findings with outliers included and excluded. Given that outliers are to be expected in large data sets, and there was no evidence to suggest they were invalid responses, we retained all cases for subsequent analyses reported in this paper.

## Results

### Descriptive statistics and preliminary analyses

Means, standard deviations, and intercorrelations for the main variables assessed in the study are presented in S1 Table. On average, participants reported that their organizations were moderately committed to pro-environmental outcomes and supporting employee autonomy, with means on both scales falling above the midpoint. For pro-environmental climate, 13% of respondents disagreed (below 2) and 25% of respondents agreed (above 4) on a 5-point scale, that their organization had pro-environmental policies and procedures reflecting a pro-environmental climate. The majority of respondents fell in the mid-level range. For provision of autonomy support, 4% of respondents disagreed (below 2) and 19% of respondents agreed (above 6) on a 7-point scale, that supervisors supported their autonomy in the workplace. On average, participants reported engaging in workplace PEB between “sometimes” and “often”, and in non-workplace PEB slightly more than “about half the time”. Examination of the correlation matrix revealed significant positive associations between (1) both work climate variables and autonomous and controlled motivation for PEB, and (2) between work climate and PEB both inside and outside the workplace. Furthermore, higher PEB motivation (both autonomous and controlled) was significantly associated with more workplace and non-workplace PEB; and the correlations between autonomous motivation and both PEBs were particularly strong. Overall, the pattern of associations suggested that both contextual factors (i.e., work climate) and individual factors (i.e., motivations) likely play a role in determining PEB inside and also outside the workplace.

### Moderated mediation analysis

Model 8 in the SPSS PROCESS macro [25] was used to test whether: (1) employee autonomy support moderated the effect of pro-environmental climate on employees’ autonomous and controlled motivation to engage in PEB, and (2) whether the effects of pro-environmental climate and autonomy support on workplace PEB and non-workplace PEB were mediated by employees’ autonomous and controlled motivation. The analyses were conducted separately for workplace and non-workplace PEB. See S1 Fig for a summary of the results from both analyses.

Beginning on the left side of S1 Fig, we first examined the effects of the two types of work climate (pro-environmental and autonomy support) and their interaction on respondents’ autonomous and controlled motivation to engage in PEB. Both main effect variables were centred at 0 prior to computing the interaction. For autonomous motivation, the main effects for pro-environmental climate and autonomy support were statistically significant, with both predicting higher levels of autonomous motivation to engage in PEB. As predicted, we also found a significant pro-environmental climate by autonomy support interaction. Examination of the conditional effects for the interaction indicated that pro-environmental climate significantly predicted autonomous PEB motivation when autonomy support was low (16^th^ percentile, *B* = .27, *SE* = .05, 99% CI = .15 to .40), moderate (50^th^ percentile, *B* = .39, *SE* = .04, 99% CI = .29 to .49), and high (84^th^ percentile, *B* = .48, *SE* = .05, 99% CI = .35 to .61), but that the effects were strongest when autonomy support was high. To further probe the interaction, we conducted a Johnson-Neyman regions of significance analysis which indicated that the effect of pro-environmental climate on autonomous PEB motivation became nonsignificant when levels of autonomy support fell below 2.59 (on a 7-point scale). The results from the Johnson-Neyman analysis suggest that positive pro-environmental climate in itself may not be sufficient to generate autonomous motivation to engage in PEB. For autonomous PEB motivation to flourish, organizations must create work climates that support both pro-environmental activities and at least a minimal level of worker autonomy.

Next, we assessed the effects of pro-environmental climate and employee autonomy support on controlled motivation to engage in PEB. In this analysis, while the main effect for pro-environmental climate was strong and significant, the main effect for autonomy support was not. As expected, the climate by autonomy support interaction effect was not significant. Consistent with our prediction, this result suggests that employees’ controlled motivation for workplace PEB is driven by the extent to which organizations have policies, protocols, and guidelines that support such initiatives. Other aspects of work climate, such as supporting worker autonomy, appear to be important for fostering an autonomously motivated workforce but have no impact on employees’ controlled motivation for PEB.

Finally, we examined the extent that autonomous and controlled motivation for PEB mediated the impact of the work climate variables (pro-environmental climate and autonomy support) on workplace PEB and also non-workplace PEB (spillover). Once again referring to S1 Fig, we find autonomous motivation for PEB significantly predicted workplace PEB, but controlled motivation did not. Examination of the tests for the indirect (mediation) effects of work climate on workplace PEB through autonomous motivation revealed significant effects at all three levels of autonomy support, with the effect sizes increasing in step with increases in autonomy support: 16^th^ percentile (*B* = .07, *SE* = .02, 99% CI = .03 to .12), 50^th^ percentile (*B* = .11, *SE* = .01, 99% CI = .07 to .15), and 84^th^ percentile (*B* = .13, *SE* = .02, 99% CI = .08 to .19). The indirect effects of the pro-environmental climate (*B* = -.01, *SE* = .01, 99% CI = -.03 to .00) and autonomy support (*B* = -.003, *SE* = .002, 99% CI = -.01 to .00) on workplace PEB through controlled motivation both failed to reach significance. Overall, this pattern of results indicates that: (1) autonomous PEB motivation is a much stronger predictor of workplace PEB than controlled PEB motivation, and (2) employees with the highest levels of autonomous PEB motivation are found in organizations with strong pro-environmental climates and provide at least moderate levels of support to encourage employee autonomy.

To assess spillover effects, we tested the same moderated mediation model as above, but replaced workplace PEB with non-workplace PEB as our dependent variable. Given that the effects of the two organizational climate variables on autonomous and controlled motivation were identical in both models, only the effects unique to the spillover analysis are discussed here.

Our spillover analysis indicated that both autonomous motivation and controlled motivation significantly predicted employee participation in non-workplace PEBs, although the effect for autonomous motivation was over twice as strong as it was for controlled motivation. Examining tests for the conditional indirect effects of the work climate variables (pro-environmental climate and employee autonomy support) on non-workplace PEB through autonomous PEB motivation, we found significant effects at all three levels of autonomy support. As was the case for workplace PEB, we found effect sizes increasing in line with increased autonomous motivation when autonomy support was low (16^th^ percentile, *B* = .11, *SE* = .03, 99% CI = .04 to .18), moderate (50^th^ percentile, *B* = .16, *SE* = .02, 99% CI = .11 to .23), and high (84^th^ percentile, *B* = .21, *SE* = .03, 99% CI = .13 to .29). Through controlled motivation, pro-environmental climate had a significant indirect effect on non-workplace PEB (*B* = .05, *SE* = .01, 99% CI = .03 to .09). As expected, there was no indirect effect of autonomy support through controlled motivation on non-workplace PEB (*B* = .01, *SE* = .01, 99% CI = -.001 to .02). Overall, our spillover analyses indicated that the effects of work climate on PEB may extend beyond the workplace, driven primarily by autonomous motivation, with a more limited role played by controlled motivation.

## Discussion

This study investigated how two aspects of work climate (i.e., pro-environmental climate and employee autonomy support) might influence employees’ motivation to engage in PEB. A central aim of the study was to assess whether workplaces with climates that support high levels of autonomous motivation for PEB might not only foster higher levels of workplace PEB, but also lead to positive spillover effects by increasing PEB outside of the workplace. We found that both workplace PEB and non-workplace PEB were higher in organizations with work climates that support both pro-environmental activity and worker autonomy, and that these climate effects could be partly explained by the extent to which workers in such environments experienced increased autonomous and controlled motivation for engaging in PEB. These findings and their implications are discussed in more detail in the sections that follow.

### Summary of main findings

Our results indicated that pro-environmental work climate predicted employee motivation, both autonomous and controlled, to engage in PEB. However, the nature of the effects varied depending on the level of autonomy provided by the organization. As hypothesized, employees working in organizations with stronger pro-environmental climates, irrespective of the level of autonomy support offered, reported higher levels of controlled PEB motivation. In contrast, employees reported higher levels of autonomous PEB motivation in organizations with strong pro-environmental climates and moderate to high levels of autonomy support. Overall, this pattern of motivational effects is consistent with the SDT model of employee motivation [26]. The combination of both pro-environmental climate and autonomy support were associated with increased autonomous motivation for PEB, whereas pro-environmental climate alone appeared to be sufficient for higher levels of controlled motivation to emerge.

We also found that employee motivation predicted workplace and non-workplace PEB in both expected and unexpected ways. As predicted, employees with higher autonomous PEB motivation engaged in more workplace PEB and also more non-workplace PEB. That is, we found a positive spillover effect to outside the workplace for workers who scored high on autonomous motivation for PEB. Consistent with SDT, employees working in organizations with strong pro-environmental climates and strong autonomy support reported higher levels of autonomous motivation and, in turn, reported engaging in more PEBs both inside and outside the workplace.

Counter to our hypotheses, higher levels of controlled motivation for PEB were associated with increased employee engagement in non-workplace PEB, but not workplace PEB. This finding runs opposite to the pattern we predicted. One possible explanation for the non-significant effect of controlled motivation on workplace PEB is that most organizations have multiple work climates that operate simultaneously, and an employee’s perception of a strong pro-environmental climate does not necessarily mean that PEB is an organization’s highest priority [6]. Work climates stipulate the workplace behaviors that employees are expected to perform and clarify the specific behaviors that will be rewarded. As such, employees with controlled PEB motivation would be likely to engage in workplace PEB only in organizations that prioritise pro-environmental climate over other potential competing climates, such as those related to safety, service, or other outcomes that an organization may value.

A second possible explanation is methodological in nature. A review of the effects in the study revealed that the motivation variables were consistently stronger predictors of non-workplace PEB than workplace PEB. Examination of the items comprising the non-workplace and workplace PEB scales suggests that many of the workplace behaviors were likely more difficult to perform (e.g., recycling chemical office waste, using narrow margins on office documents, and pointing out co-workers un-ecological behavior) than the non-workplace ones (e.g., recycling newspapers and bottles, avoiding littering, and reusing plastic containers). In the presence of such difficulties, one would expect the predictive effects of motivation on workplace PEB to be smaller than the effects of motivation on non-workplace PEB, a pattern that is consistent with the correlations presented in S1 Table. The correlations also reveal that controlled motivation was significantly correlated with both workplace and non-workplace PEB, and only when both motivational factors were entered together in the regression analysis did the effect of controlled motivation on workplace PEB become nonsignificant. Thus, it seems plausible that controlled motivation predicts both workplace and non-workplace PEBs, but that the workplace effects in this study were weaker due to the greater difficulty of the items. Future research should systematically explore the impact of item difficulty on the magnitude of motivational effects on PEB across different contexts.

### Practical implications

Our results indicate that autonomous PEB motivation is a much stronger predictor of workplace PEB than controlled PEB motivation. This highlights the importance of considering the reasons why employees engage in PEB when designing workplace interventions to increase PEB. We found that employees with the highest levels of autonomous PEB motivation are found in organizations that support both pro-environmental activity and workers’ autonomy, indicating that building an eco-workforce requires organizations to not only have strong pro-environmental policies and procedures but to also support their workers’ autonomy.

An optimal workplace, then, would provide the right conditions for engaging in PEBs in the first place. This might involve increasing employees’ feelings of autonomy over their own environmental behaviors by, for example, having control over their own electricity use, recycling behavior, etc. This would encourage employees to explore environmental activities they find interesting and challenging, which has the potential to support the internalization of environmental values, providing the motivational foundation for additional pro-environmental behaviors both inside and outside the workplace [3].

### Limitations and future research

This study had several limitations that should be considered when interpreting our findings. First, our study relies on self-reported data provided by employees recruited from a non-probability sample. Although we employed a large, diverse national sample, findings cannot presume to be generalizable to the broader Australian population or to other countries. To evaluate the robustness of our findings, we recommend additional studies using a variety of samples, including those from other countries and cultures and recruited in ways other than through an online panel. We also recommend collecting information using more objective measures for work climate (e.g., independent analysis of organizational policies) and employee PEBs (e.g., data from waste audits and energy monitoring systems).

A second important limitation of this study is that it employed a correlational research design. Although mediation analysis implies a causal explanation [25], in the present study, it should not be used to make strong causal claims. Although we identified several significant indirect effects of work climate on employees’ PEB through two motivational constructs, at best we can only conclude that this pattern of results is consistent with a causal path. It is possible that some of the associations between climate, motivation, and behavior may in fact be bi-directional. For example, individuals with strong autonomous motivation for PEB may be more likely to perceive their organizations as having green climates, and engaging in PEB may reinforce pro-environmental motivation. Controlling for participants’ ecological worldviews should partially control for these reciprocal effects. Nevertheless, future research using experimental research designs in which variables are experimentally manipulated are necessary to make stronger claims about direction of causality. Future research should also examine other variables such as needs, norms, and self-identity not included in the current study that may explain relationships between work climate and PEB.

A major outstanding challenge for academics and practitioners is evaluating actual changes in PEB and also measuring sustained PEB resulting from a particular intervention. Understanding how to change and permanently shift people’s behavior is essential for interventions to be supported within businesses and by government. Thus, we recommend collecting information on employee PEB at multiple time points to test the stability of PEB associated with different interventions.

## Conclusion

In this study, we investigated the effects of two types of work climate (pro-environmental climate and organizational support for employee autonomy) on employees’ propensity to engage in PEB both at work and outside the workplace. Testing a model based on SDT, we also investigated whether these effects were mediated by two types of employee motivation: controlled and autonomous. We found that in workplaces with stronger pro-environmental climates and at least moderate levels of autonomy support, employees reported higher levels of autonomous motivation to engage in PEB. In turn, employees with higher levels of autonomous motivation engaged in more PEBs, both inside and outside the workplace. Controlled motivation played a much more limited role in predicting employee PEBs in workplace and non-workplace settings. Overall, our findings suggest work climates that support pro-environmental actions and employee autonomy not only foster PEBs within the workplace but also lay the foundation for PEBs in other non-workplace settings.

## Supporting information

S1 Fig Moderated mediation model showing (1) autonomy support moderating the effects of pro-environmental climate on autonomous PEB motivation, and (2) autonomous PEB motivation mediating the effects of the work climate variables on workplace PEB and non-workplace PEB. Participants’ ecological worldview, as assessed by the NEP, was included as a covariate in the model to control for common method bias, as well as any pre-existing differences in pro-environmental values and attitudes. Values on pathways represent unstandardized regression weights (**p < .01, ***p < .001). Model fit indices: *R* = 0.60, *R*^*2*^ = 0.36, *F* = 76.62*** for workplace PEB and *R* = 0.68, *R*^*2*^ = 0.46, *F* = 113.73*** for non-workplace PEB.
(TIF)

S1 Table Zero-order correlations and descriptive statistics. *N* = 818. ** *p* < .01.
(TIF)
