# Supplementary figures and images for: Can work climate foster pro-environmental behavior inside and outside of the workplace?
Source: PLoS One. 2019 Oct 10;14(10):e0223774. doi: 10.1371/journal.pone.0223774 (PMC6786752; doi:10.1371/journal.pone.0223774)

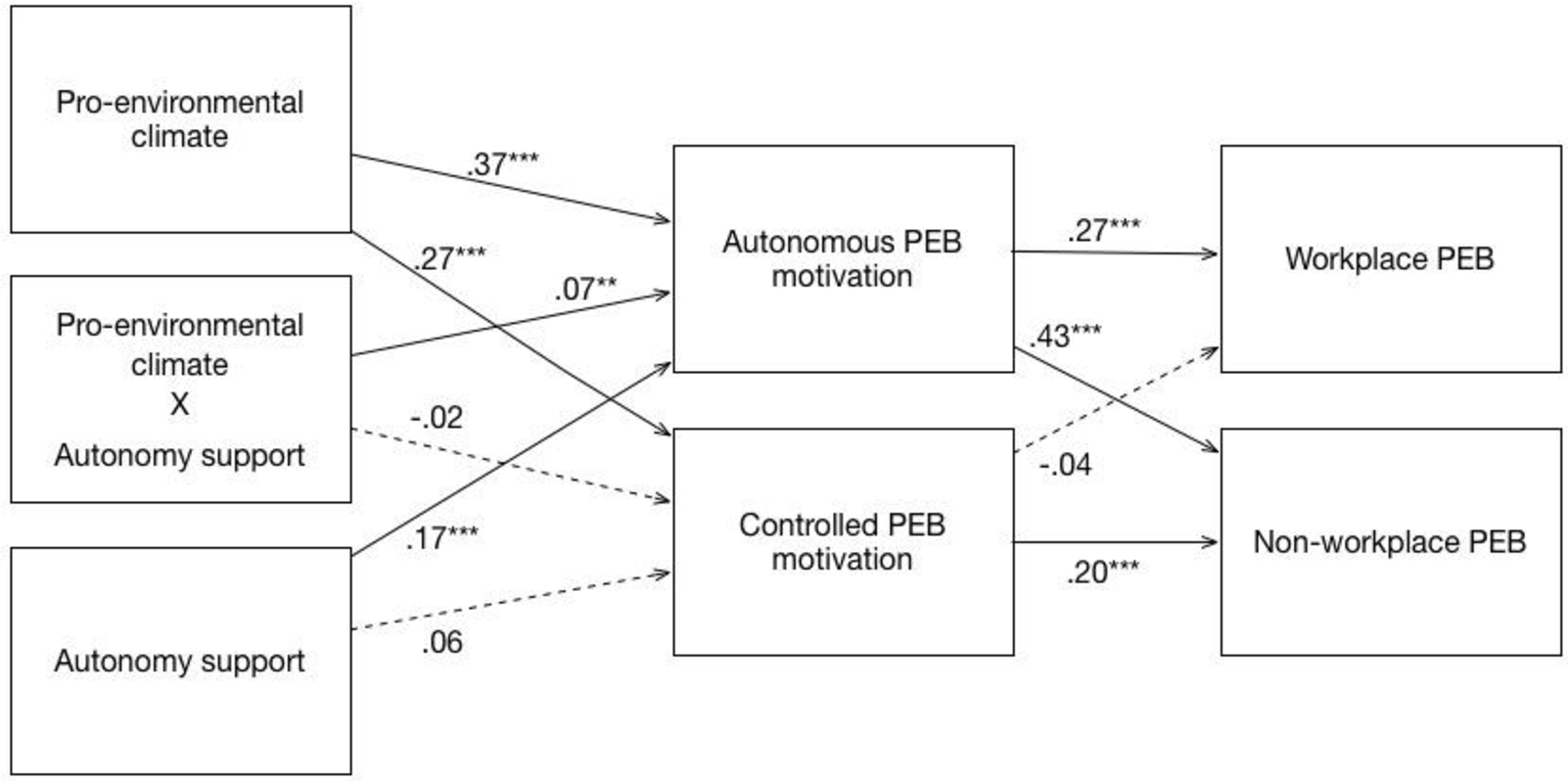

Supplement: S1 Fig — Participants’ ecological worldview, as assessed by the NEP, was included as a covariate in the model to control for common method bias, as well as any pre-existing differences in pro-environmental values and attitudes. Values on pathways represent unstandardized regression weights (**p < .01, ***p < .001). Model fit indices: R = 0.60, R2 = 0.36, F = 76.62*** for workplace PEB and R = 0.68, R2 = 0.46, F = 113.73*** for non-workplace PEB. (TIF) [file pone.0223774.s001.tif]

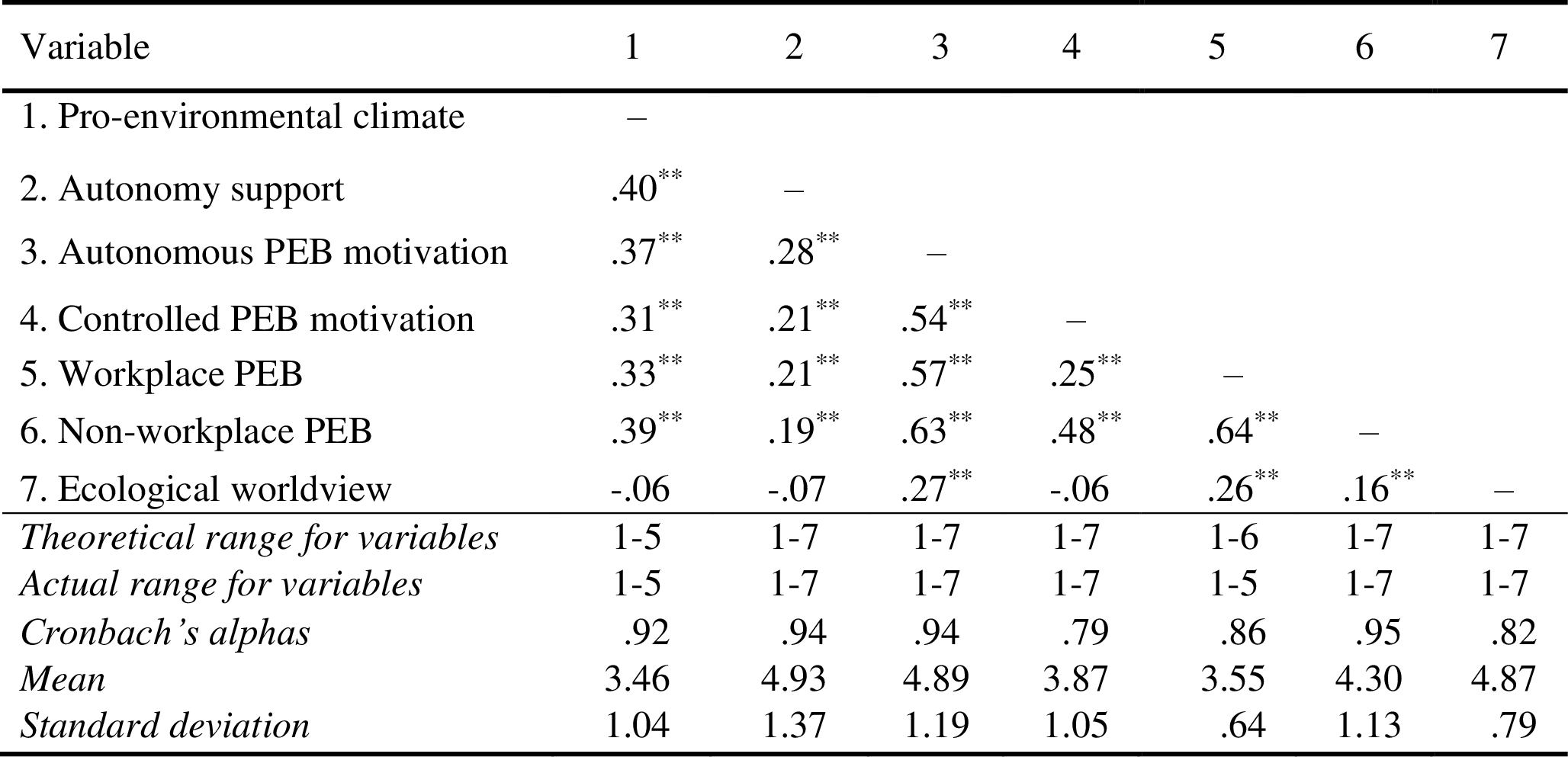

Supplement: S1 Table — N = 818. ** p < .01. (TIF) [file pone.0223774.s002.tif]
